# Cannabinol Inhibits Cellular Proliferation, Invasion, and Angiogenesis of Neuroblastoma via Novel miR-34a/tRiMetF31/PFKFB3 Axis

**DOI:** 10.3390/cancers14081908

**Published:** 2022-04-10

**Authors:** Bo Wang, Dongping Li, Viktoriia Cherkasova, Marta Gerasymchuk, Aru Narendran, Igor Kovalchuk, Olga Kovalchuk

**Affiliations:** 1 Department of Biological Sciences, University of Lethbridge, Lethbridge, AB T1K3M4, Canada; bo.wang5@uleth.ca (B.W.); dongping.li@uleth.ca (D.L.); viktoriia.cherkasova@uleth.ca (V.C.); marta.gerasymchuk@uleth.ca (M.G.); 2 Arnie Charbonneau Cancer Institute, University of Calgary, Calgary, AB T2N 4Z6, Canada; a.narendran@ucalgary.ca

**Keywords:** cannabinol, neuroblastoma, proliferation, angiogenesis, miR-34a/tRiMetF31/PFKFB3 axis

## Abstract

**Simple Summary:**

The prognosis of high-risk neuroblastoma is poor due to its high relapse rate. To date, no effective treatment for this disease has been developed. In this study, we utilized two neuroblastoma cell lines (IMR-5 and SK-N-AS) as a model system to explore the effects of cannabinol (CBN) on neuroblastoma and elucidate the potential mechanisms of action. We reveal an inhibitory role of CBN on neuroblastoma cell proliferation, invasion, and angiogenesis through miR-34a-mediated targeting. We identified 6-phosphofructo-2-kinase/fructose-2,6-biphosphatase 3 (PFKFB3) as a direct target of a novel 31 nt tRNA_i_^Met^ fragment tRiMetF31 generated from miR-34a-guided cleavage, highlighting the crucial role of the miR-34a/tRiMetF31/PFKFB3 axis in CBN-mediated suppression in neuroblastoma biology.

**Abstract:**

High-risk neuroblastoma is an aggressive pediatric tumor. Despite great advances in neuroblastoma therapy and supportive care protocols, no curative treatment is available for most patients with this disease. Here, we uncover that CBN attenuated the cell proliferation, invasion, and angiogenesis of neuroblastoma cell lines in a dose-dependent manner via the inhibition of the AKT pathway and the upregulation of miR-34a that targets E2F1. Both miR-34a and a 31-nt tRNA_i_^Met^ fragment (tRiMetF31) derived from miR-34a-guided cleavage were downregulated in 4 examined neuroblastoma cell lines inversely correlated with the levels of its direct target, the PFKFB3 protein. Moreover, ectopic tRiMetF31 suppressed proliferation, migration, and angiogenesis in the studied neuroblastoma cell lines. Conversely, tRiMetF31 knockdown promoted PFKFB3 expression, resulting in enhanced angiogenesis. Our findings reveal a suppressive role of CBN in neuroblastoma tumorigenesis, highlighting a novel and crucial miR-34a tumor suppressor network in CBN’s antineuroblastoma actions.

## 1. Introduction

Neuroblastoma is globally the most common extracranial solid tumor in the pediatric population, accounting for about 10% of all tumors in this age group [1]. As a highly heterogeneous tumor, neuroblastoma carries significant morbidity and mortality, accounting for 15% of all cancer-related death in this population [2]. According to the current Children’s Oncology Group (COG) risk stratification system, patients with neuroblastoma can be classified into low-, intermediate-, and high-risk categories predictive of relapse [3]. Clinically, children with high-risk neuroblastoma generally display a high relapse rate (50–60%) and poor prognosis, and disease-free survival is less than 30% [3,4]. It is hard to effectively treat high-risk neuroblastoma. Although the long-term survival of those patients is improved by utilizing intensive multimodal therapy, serious side effects on children’s endocrine system and growth were reported [5]. Therefore, approaches on novel therapies and drugs are urgently needed.

Neuroblastoma is a complex and heterogeneous disease. So far, genetic, epigenetic, and molecular biological mechanisms have been demonstrated to be involved in the growth, survival and metastasis of the malignant cells. Germline mutations of anaplastic lymphoma kinase (*ALK*) and paired-like homeobox 2B (*PHOX2B*) genes are pivotal predisposition factors in hereditary neuroblastoma [6,7,8]. Recently, potentially pathogenic germline variants of *ALK*, *CHEK2*, *PINK1* and *BARD1* genes were discovered in high-risk neuroblastoma [9], including the ALK p.Arg1275Gln variant, which is frequently seen in familial neuroblastoma [6,7]. Dysregulated histone modifications are also linked to carcinogenesis of neuroblastoma. Histone deacetylase 8 (HDAC8) was overexpressed in metastasized stage 4 neuroblastomas [10], which correlated with poor prognosis in addition to stage 4S disease. Evidence demonstrated a contributing role of numerous miRNAs in neuroblastoma tumorigenesis and drug resistance [11,12,13]. Signaling pathways are essential for cells to modulate their function in response to environmental stimuli and to communicate with surrounding cells, play a crucial role in all biological and pathological processes. Signaling pathways are frequently altered in malignancies [14]; consequently, altered pathways may contribute to the development of oncogenic hallmarks [15]. The PI3K/AKT/mTOR pathway is activated in neuroblastoma tissue, and proliferative signaling is attenuated by inhibitors both in vitro and in vivo [16]. Polo-like kinase 4, a key molecule regulating centriole replication, promotes neuroblastoma tumorigenesis and metastasis through the PI3K/AKT pathway in cell lines and animal models [17], and the knockdown of AKT induced neuroblastoma apoptosis under hypoxia [18]. The hedgehog and NF-κB pathways may also play a role in neuroblastoma development [19,20]. Established neuroblastoma cell lines represent a highly validated experimental tool to understand the pathogenesis and therapeutic vulnerabilities of neuroblastoma, as they represent the molecular and growth-regulatory aberrations seen in clinical specimens [21].

The endocannabinoid system (ECS) is a complex signaling pathway in which endocannabinoids exert their biological effects through binding to cannabinoid receptors (CBRs), mainly CBR1 (also known as CB1) and CBR2 (also known as CB2), which are members of the G-protein-coupled receptor family. In addition to endocannabinoids, two other cannabinoids, phytocannabinoids, mainly produced by *Cannabis sativa*, and synthetic cannabinoids can also impact the biological function of human cells via the ECS system due to the structural similarity to endocannabinoids [22]. To date, more than 120 phytocannabinoids have been identified, and the most abundant constituents are cannabichromene (CBC), cannabidiol (CBD), cannabidivarin (CBDV), cannabigerol (CBG), cannabinol (CBN), cannabivarin (CBV), Δ^9^-tetrahydrocannabinol (Δ^9^-THC), Δ^8^-tetrahydrocannabinol (Δ^8^-THC), and Δ^9^-tetrahydrocannabivarin (THCV) [23]. Accumulating evidence shows the anticancer and anti-inflammatory effects of cannabinoids [22,24]. However, the inhibitory effect of CBN on neuroblastoma tumorigenesis remains unknown.

In this study, we uncover that, in two neuroblastoma cell lines IMR-5 (wild-type p53) and SK-N-AS (mutant p53), CBN attenuates cell proliferation, angiogenesis, and invasion in a dose-dependent manner via inhibiting the AKT pathway and upregulating miR-34a, which targets E2F1. We demonstrate that a 31-nt tRNA_i_^Met^ fragment (tRiMetF31) derived from miR-34a-guided cleavage may function as a tumor suppressor to inhibit cell proliferation and angiogenesis through directly targeting PFKFB3, providing novel insight into the suppressive role of the miR-34a tumor suppressor network in neuroblastoma progression.

## 2. Results

### 2.1. CBN Inhibits Cell Proliferation, Invasion, and Angiogenesis of Neuroblastoma via Inhibiting AKT Pathway and Upregulating miR-34a

**Figure 1:**
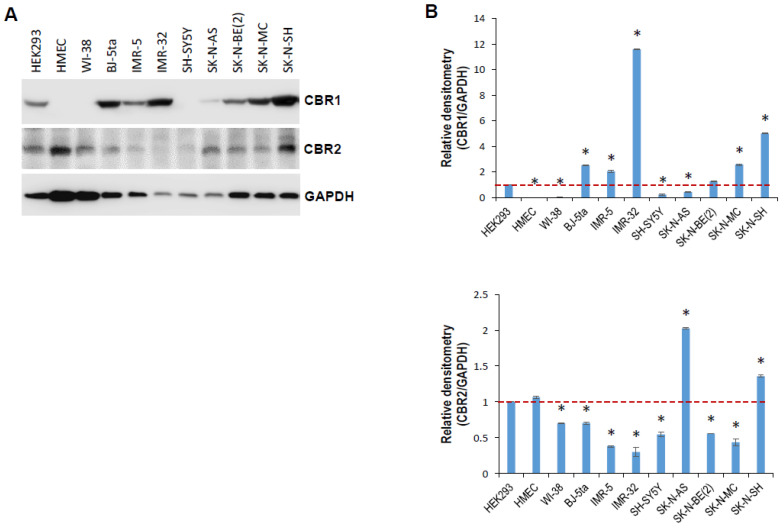
Expression of cannabinoid receptors CBR1 and CBR2 in normal and neuroblastoma cell lines. (**A**) Whole cellular lysates were prepared from indicated normal and neuroblastoma cell lines, and subjected to Western blot analysis using antibodies against CBR1 and CBR2 as described in Section 4; GAPDH served as a loading control. (**B**) Relative densitometry was measured using ImageJ, calculated as a ratio to GAPDH, and expressed as mean ± SD for three independent measurements. *, *p* < 0.05. Original western blot data is shown in Figure S3.

**Figure 2:**
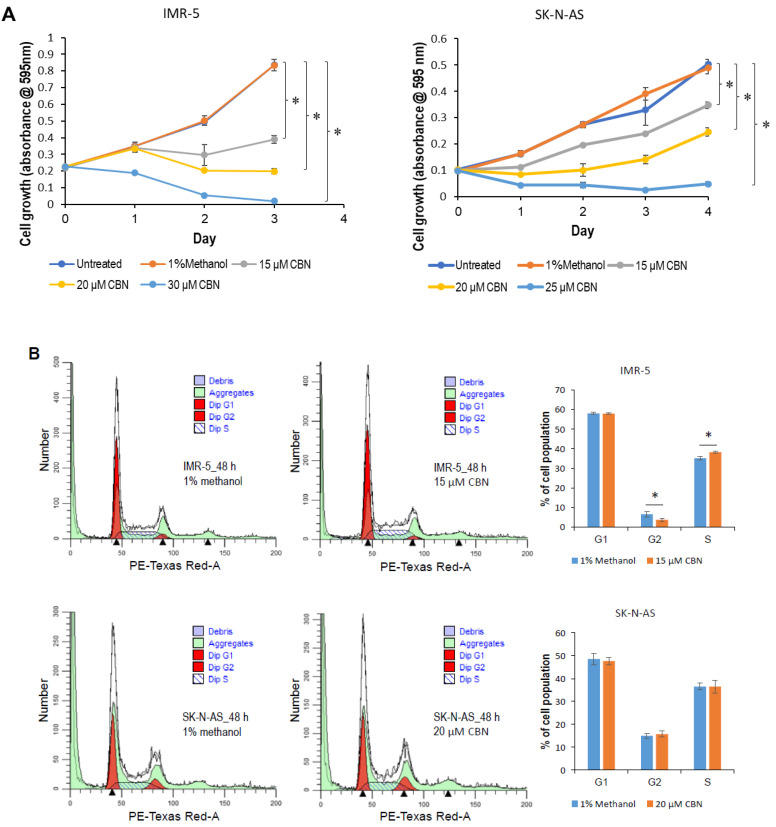

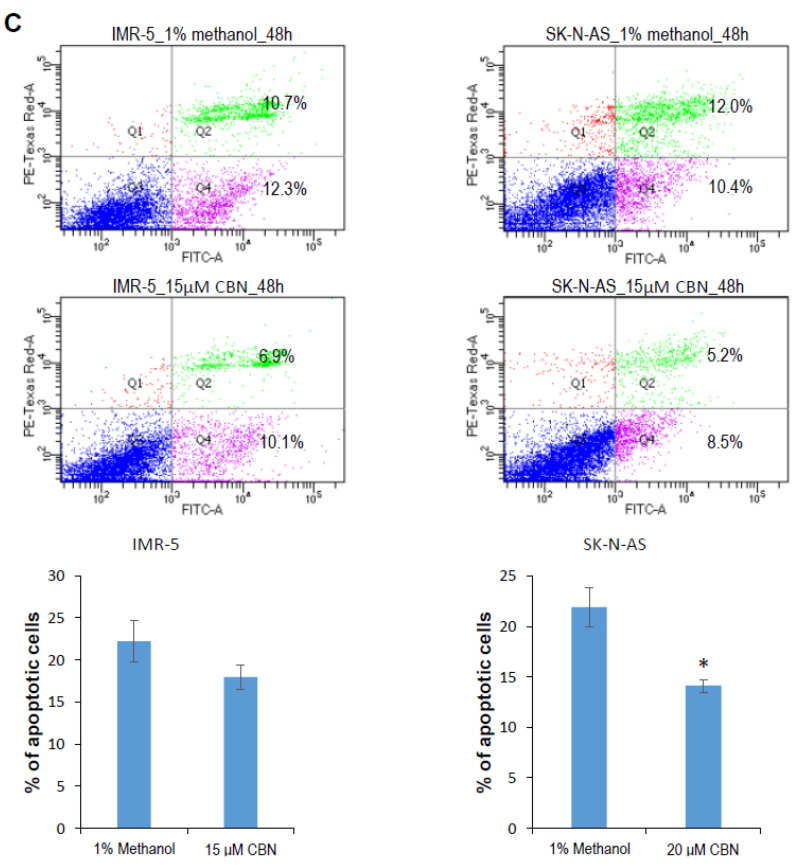

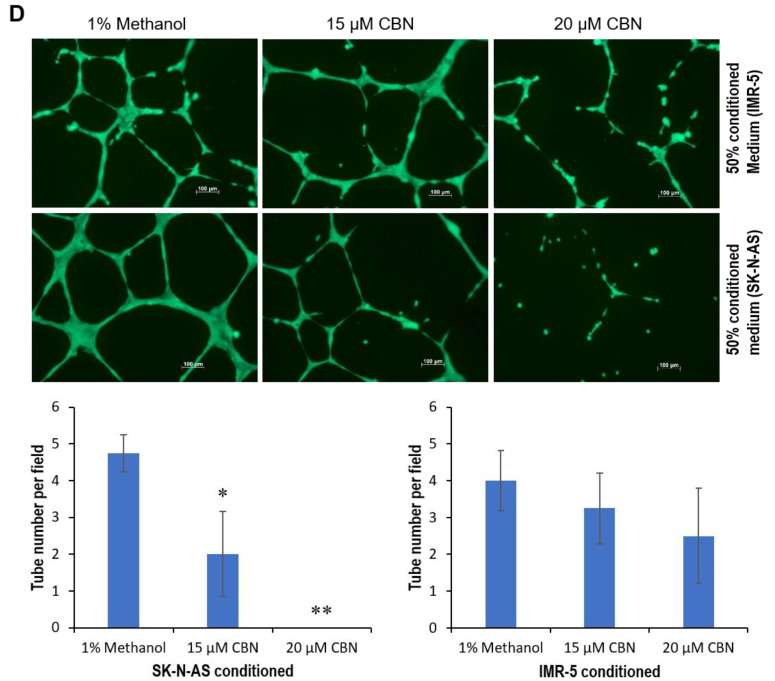

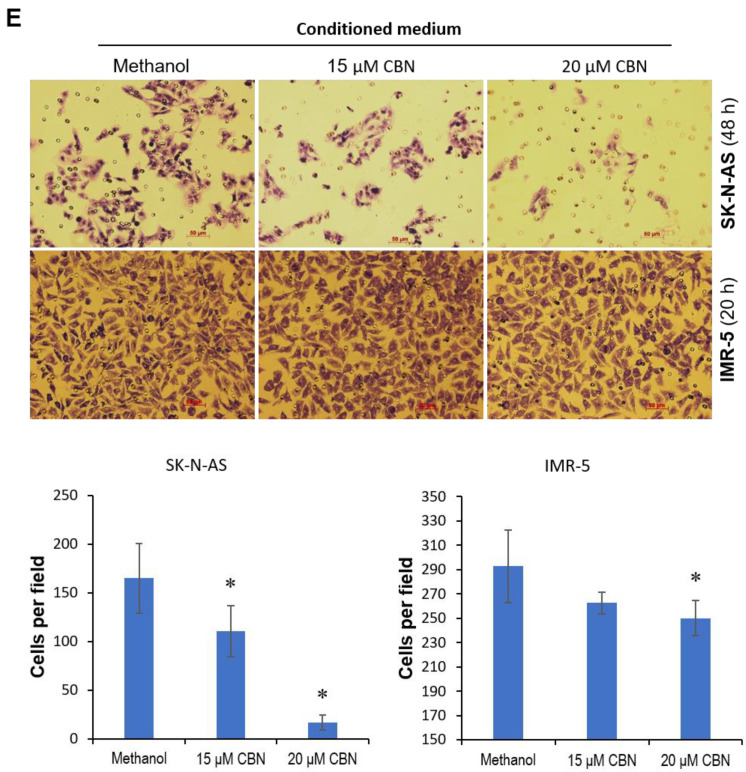

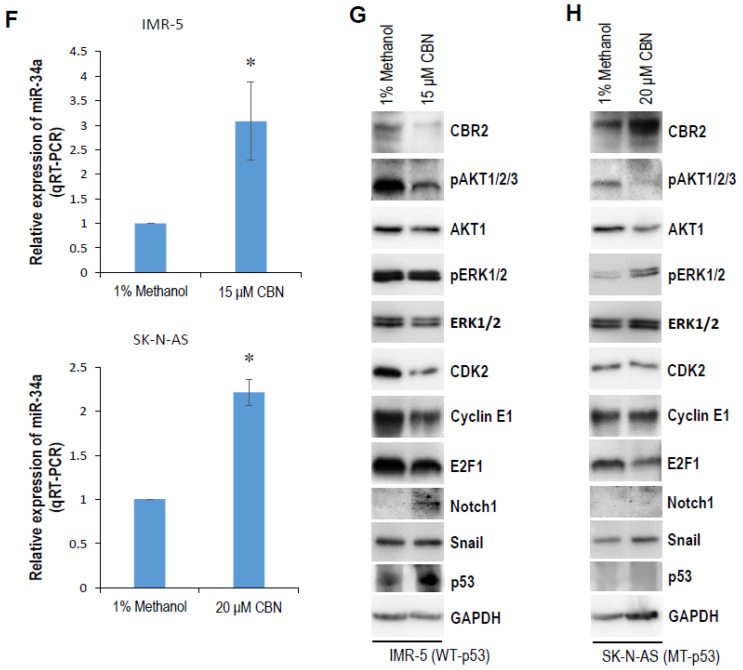

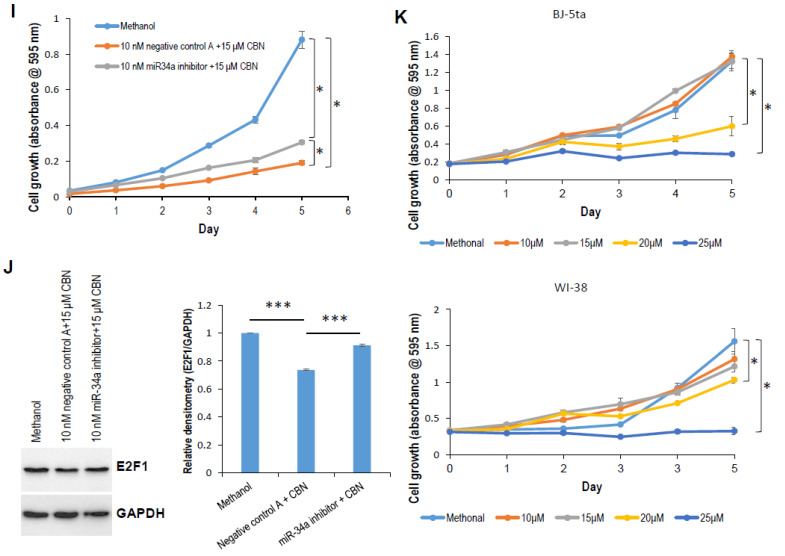
Anti-neuroblastoma effect of CBN via inhibition of AKT pathway and transactivation of miR-34a. (**A**) 1000 IMR-5 cells per well and 2000 SK-N-AS cells per well were plated in 96-well plates. At 24 h after plating, cells were treated with the indicated concentration of CBN, and MTT assay was performed using Cell Proliferation Kit I as described in Section 4. (**B**,**C**) IMR-5 and SK-N-AS cells grown to 85% confluency were exposed to either 15 or 20 μM CBN. At 48 h after treatment, cells were harvested for cell cycle and apoptosis analyses. (**D**) Tube formation assay was carried with 50% of conditioned medium from either IMR-5 or SK-N-AS cells exposed to either 15 or 20 µM CBN as detailed in Section 4; representative images were taken using a fluorescence microscope (100×). Data were expressed as mean ± SD for five images. (**E**) Cell invasion assay was performed using 33% of conditioned medium from either IMR-5 or SK-N-AS cells exposed to either 15 or 20 µM CBN as detailed in Section 4; representative images were taken under inverted microscope (200×). Data were expressed as mean ± SD for five images. (**F**) IMR-5 and SK-N-AS cells grown to 85% confluency were exposed to either 15 or 20 μM CBN. At 48 h after treatment, total RNA was isolated and subjected to qRT-PCR analysis using hsa-miR-34a primer set. (**G**,**H**) IMR-5 and SK-N-AS cells grown to 85% confluency were exposed to either 15 or 20 μM CBN. At 48 h after treatment, whole cellular lysates were prepared and subjected to Western blotting with antibodies to CBR2, pAKT1/2/3, AKT1, pERK1/2, ERK1/2, CDK2, cyclin E1, E2F1, notch1, snail, and p53, GAPDH served as a loading control. (**I**) IMR-5 cells grown to 80% confluency were transfected with either 10 nM LNA miR-34a Power Inhibitor or 10 nM negative control A. At 24 h after transfection, 2000 cells per well were plated in 96-well plates and exposed to 15 μM CBN; MTT assay was carried out as described in Section 4. (**J**) IMR-5 cells grown to 80% confluency were transfected with either 10 nM LNA miR-34a Power Inhibitor or 10 nM negative control A. At 24 h after transfection, cells were exposed to 15 µM CBN. At 48 h after exposure, whole cellular lysates were prepared and subjected to Western blotting with antibody against E2F1; GAPDH served as a loading control. (**K**) 3000 BJ-5ta cells per well and 5000 WI-38 cells per well were plated in 96-well plates. At 24 h after plating, cells were exposed to the indicated concentration of CBN, and the MTT assay was performed using Cell Proliferation Kit I as detailed in Section 4. *, *p* < 0.05; ** *p* < 0.01, ***, *p* < 0.001. Original western blot data is shown in Figure S3.

To establish a model system in which the role of CBN in neuroblastoma biology can be explored, we examined the expression levels of cannabinoid receptors, including CBR1 and CBR2, in normal and neuroblastoma cell lines. Western blot analysis showed that CBR1 and CBR2 were differentially expressed in the indicated normal and neuroblastoma cell lines (Figure 1A,B). We selected p53 wild-type IMR-5 (expressing high CBR1 and low CRB2) and p53 mutant SK-N-AS (expressing low CBR1 and high CBR2) neuroblastoma cell lines as a model system. The half maximal inhibitory concentration (IC50) of CBN was measured and calculated, 20.23 and 29.88 µM for IMR-5 and SK-N-AS (Figure S1), respectively. We then determined effect of CBN on cell proliferation. MTT assay indicated that CNB profoundly attenuated cell proliferation of both neuroblastoma cell lines in a dose-dependent manner (Figure 2A). The low dose (15 µM) displayed an inhibitory effect, while the high dose (30 µM for IMR-5, 25 µM for SK-N-AS) showed a killing effect (cell death > cell proliferation). CBN induced an S-phase arrest in IMR-5 cells, whereas attenuated apoptosis in SK-N-AS cells (Figure 2B,C). Furthermore, tube formation and cell invasion assays showed that the conditioned medium from SK-N-AS cells treated with 15 or 20 μM CBN profoundly attenuated angiogenesis and invasion (Figure 2D,E), while that were moderately inhibited by the conditioned medium from IMR-5 cells treated with same dose of CBN.

CBD upregulates miR-34a [25], a key player in the p53 tumor suppressor network [26], in LPS-stimulated microglia cells. Considering the structural similarity between CBD and CBN, we examined the effect of CBN on miR-34a expression. Quantitative RT-PCR (qRT-PCR) showed a significant induction of miR-34a in IMR-5 and SK-N-AS cells in response to CBN (Figure 2F). To understand molecular mechanisms mediating the antineuroblastoma effect of CBN, we looked at changes in signaling pathways, S-phase regulators, and miR-34a targets. Western blot analysis indicated that CDK2, cyclin E1, E2F1, and phosphorylated AKT1/2/3 were downregulated in both cell lines in response to CBN (Figure 2G,H). CBN inhibited CBR2 expression, and promoted the expression of notch1 and p53 in IMR-5 cells (Figure 2G). However, in SK-N-AS cells, CBN increased levels of CBR2 and phosphorylated ERK1/2, and suppressed AKT1 expression (Figure 2H). Snail expression was not affected by CBN in either line.

To further establish the key role of miR-34a in CBN antineuroblastoma signaling, we performed a rescue study using a miR-34a inhibitor. The inhibition of miR-34a restored E2F1 expression and cell proliferation that had been suppressed by CBN (Figure 2I,J). To examine whether CBN functions differentially between normal and tumor cells, we determined the effect of CBN on normal cell proliferation. MTT assay showed that low-dose (15 μM) CBN had no effect, while high-dose (25 μM) CBN inhibited the proliferation of normal cell lines tested (Figure 2K). Taken together, these results suggest that CBN suppresses the cellular proliferation, invasion, and angiogenesis of neuroblastoma via inhibiting the AKT pathway and enhancing tumor suppressor miR-34a expression. Our findings also suggest that, in response to CBN, normal cells are not as sensitive as the examined neuroblastoma cells are.

### 2.2. tRiMetF31 Is Downregulated, whereas Its Direct Target PFKFB3 Is Overexpressed in Neuroblastoma Cells

**Figure 3:**
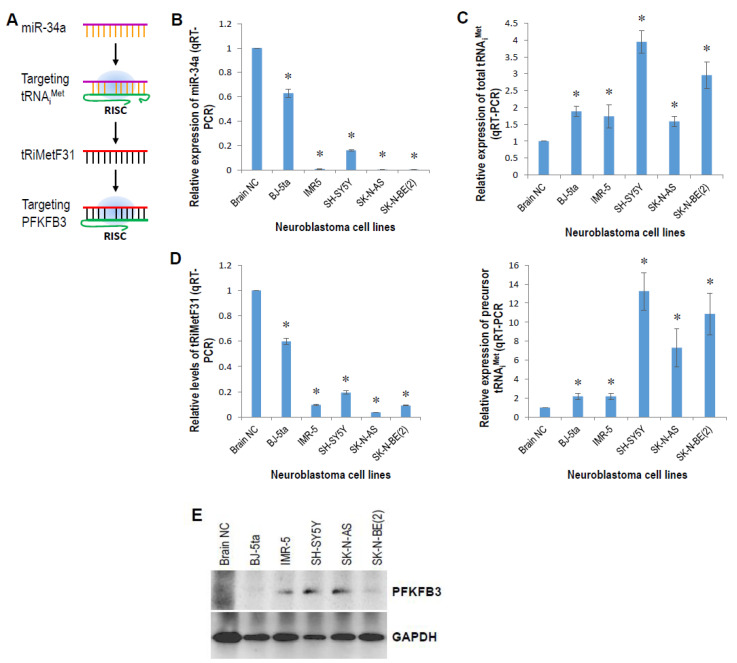
Inverse correlation between tRiMetF31 and its target PFKFB3. (**A**) Diagram of tRiMetF31 targeting PFKFB3. (**B**–**D**) Total RNA was isolated from BJ-5ta, IMR-5, SH-SY5Y, SK-N-AS, and SK-N-EB(2) cell lines and normal brain tissue, and subjected to qRT-PCR analysis of miR-34a, tRiMetF31, total tRNA_i_^Met^ and tRNA_i_^Met^ precursor as detailed in Section 4. (**E**) Whole cellular lysates were prepared from BJ-5ta, IMR-5, SH-SY5Y, SK-N-AS and SK-N-EB(2) cell lines and normal brain tissue and subjected to Western blotting with antibody to PFKFB3, GAPDH served as a loading control. *, *p* < 0.05. Original western blot data is shown in Figure S3.

A recent scientific breakthrough in our lab was the discovery of miR-34 directly targeting tRNA_i_^Met^, triggering cell cycle arrest and apoptosis, eventually leading to the proliferative inhibition of breast cancer cells [27]. RNA-Seq analysis further identified a 31-nt tRNA_i_^Met^-derived piR_019752-like fragment (tRiMetF31) from miR-34a-guided cleavage (unpublished data). Luciferase assay validated that tRiMetF31 directly targets PFKFB3 (Figure 3A), a glycolytic activator highly expressed in proliferating tissue. Here, we measured the expression levels of miR-34a, the tRNA_i_^Met^ precursor, and tRiMetF31 in neuroblastoma cell lines, and correlated tRiMetF31 levels with PFKFB3 levels. qRT-PCR showed that miR-34a and tRiMetF31 were downregulated, while total tRNA_i_^Met^ and precursor tRNA_i_^Met^ were upregulated in 4 examined neuroblastoma cell lines (Figure 3B–D). miR-34a and tRiMetF31 were decreased, whereas total tRNA_i_^Met^ and precursor tRNA_i_^Met^ were increased in normal BJ-5ta cells (Figure 3B–D). As expected, Western blot analysis indicated that PFKFB3 was overexpressed in 4 neuroblastoma cell lines (Figure 3E), and its expression levels were closely correlated with that of tRiMetF31 (Figure 3D).

### 2.3. tRiMetF31 Inhibits Cell Proliferation and Induces Cell Cycle Arrest

**Figure 4:**
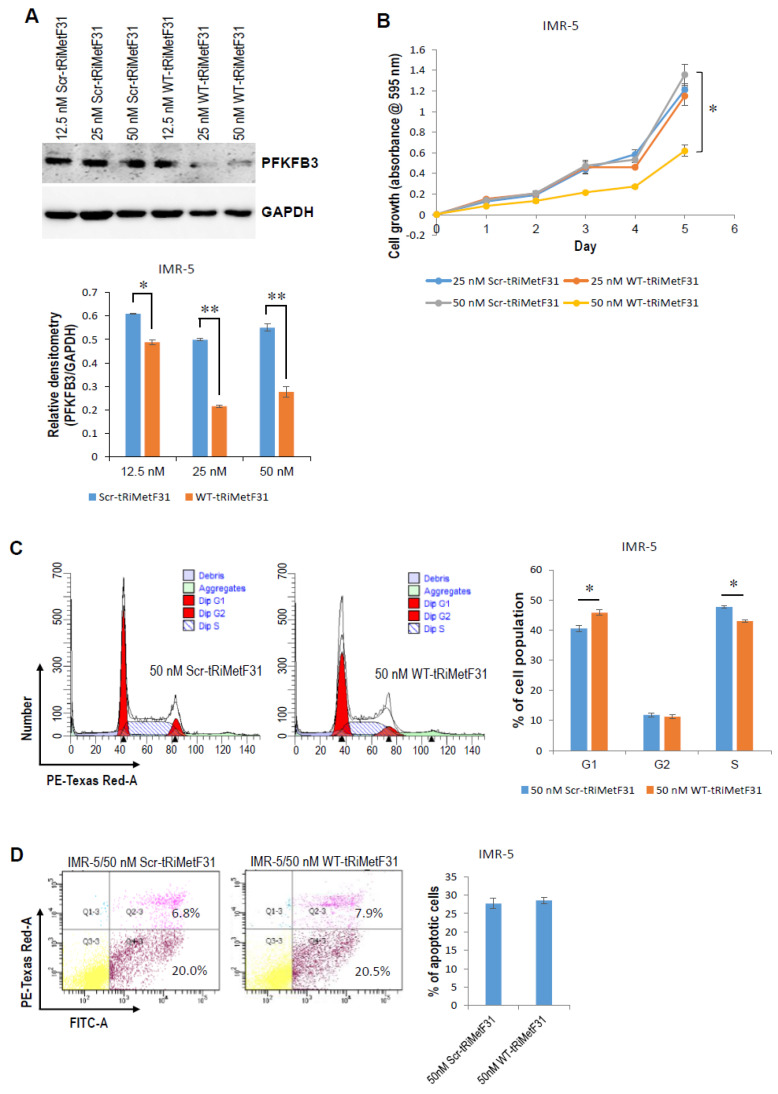
tRiMetF31 inhibits proliferation and induces G1 cell cycle arrest of IMR-5 cells. (**A**) IMR-5 cells were transfected with 12.5, 25, or 50 nM of either WT-tRiMetF31 or Scr-tRiMetF31. At 72 h after transfection, whole cellular lysates were prepared and subjected to Western blot analysis using antibody against PFKFB3, and GAPDH served as a loading control. Relative densitometry was measured using ImageJ, calculated as a ratio to GAPDH, and expressed as mean ± SD for three independent measurements. (**B**) IMR-5 cells were transfected with 25 or 50 nM of either WT-tRiMetF31 or Scr-tRiMetF31. At 24 h after transfection, cells were replated in 96-well plates, and MTT assay was performed as described in Section 4. (**C**,**D**) IMR-5 cells were transfected with 50 nM of either WT-tRiMetF31 or Scr-tRiMetF31. At 72 h after transfection, cells were harvested for cell cycle and apoptosis analyses as detailed in Section 4. *, *p* < 0.05; **, *p* < 0.01. Original western blot data is shown in Figure S3.

**Figure 5:**
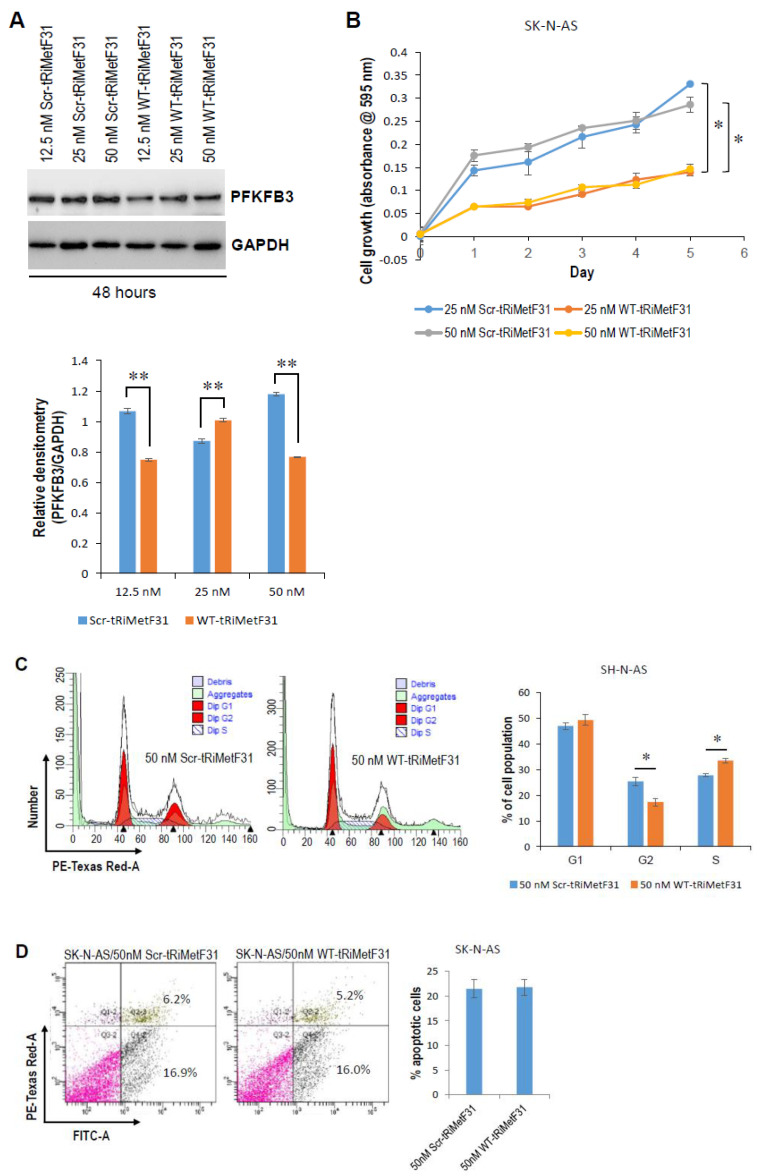
tRiMetF31 inhibits proliferation and induces S-phase cell cycle arrest of SK-N-AS cells. (**A**) SK-N-AS cells were transfected with 12.5, 25, or 50 nM of either WT-tRiMetF31 or Scr-tRiMetF31. At 48 h after transfection, whole cellular lysates were prepared and subjected to Western blotting with antibody to PFKFB3, and GAPDH served as a loading control. Relative densitometry was measured using ImageJ, calculated as a ratio to GAPDH, and expressed as mean ± SD for three independent measurements. (**B**) SK-N-AS cells were transfected with 25 or 50 nM of either WT-tRiMetF31 or Scr-tRiMetF31. At 24 h after transfection, cells were replated in 96-well plates, and MTT assay was performed as detailed in Section 4. (**C**,**D**) SK-N-AS cells were transfected with 50 nM of either WT-tRiMetF31 or Scr-tRiMetF31. At 48 h after transfection, cells were harvested for cell cycle and apoptosis analyses as detailed in Section 4. *, *p* < 0.05; **, *p* < 0.01. Original western blot data is shown in Figure S3.

To examine whether tRiMetF31 affects cell proliferation, cell cycle, and apoptosis, we transiently transfected neuroblastoma cells with either wild-type (WT) or scrambled (Scr) tRiMetF31. Western blot analysis indicated that 25 and 50 nM WT-tRiMetF31 profoundly attenuated the PFKFB3 expression of IMR-5 cells (Figure 4A), and 50 nM WT-tRiMetF31 significantly suppressed IMR-5 cell proliferation and induced G1 arrest (Figure 4B,C, *p* < 0.05), while it had no effect on apoptosis (Figure 4D). In another neuroblastoma line, SK-N-AS, Western blot analysis showed that 12.5 and 50 nM WT-tRiMetF31 inhibited PFKFB3 expression (Figure 5A). MTT assay indicated that both 25 and 50 nM WT-tRiMetF31 profoundly suppressed SK-N-AS cell proliferation, although 25 nM WT-tRiMetF31 did not influence PFKFB3 expression (Figure 5B). In addition, 50 nM WT-tRiMetF31 induced SK-N-AS S-phase cell cycle arrest (Figure 5C), whereas it had no effect on apoptosis (Figure 5D). Interestingly, although different tested concentrations of WT-tRiMetF31 apparently attenuated PFKFB3 expression, they did not affect the proliferation of IMR-32 neuroblastoma cells (Figure S2). These results suggest that tRiMetF31 inhibits neuroblastoma cell proliferation and induces cell cycle arrest via targeting PFKFB3, and the biological effect may depend on cell contents.

### 2.4. tRiMetF31 Suppresses Migration and Angiogenesis

**Figure 6:**
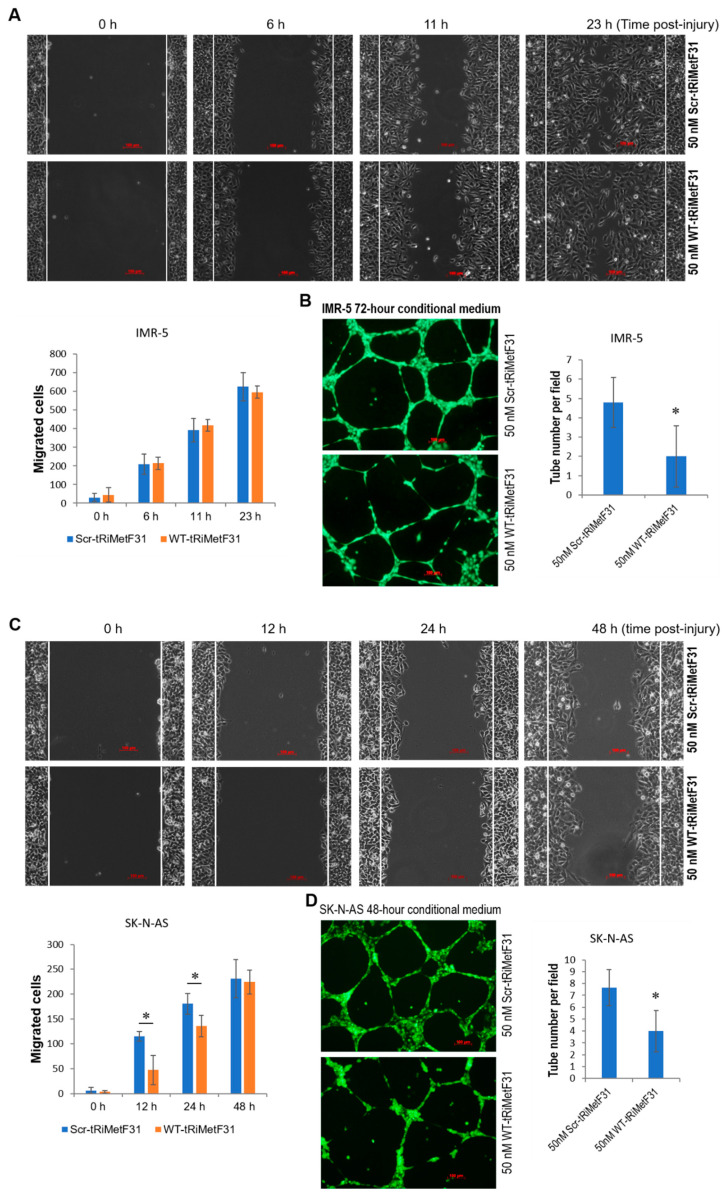

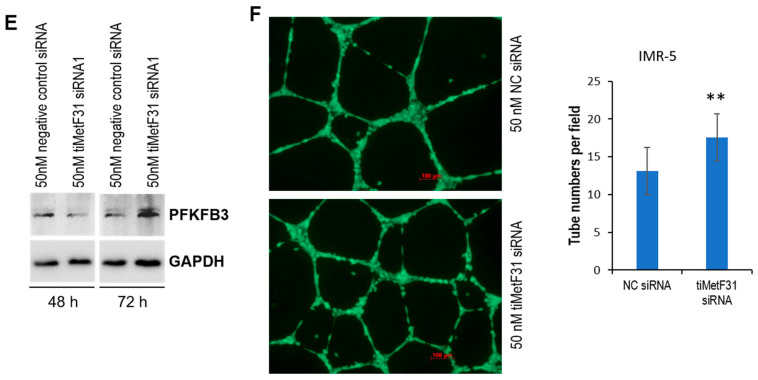
tRiMetF31 inhibits migration and angiogenesis of neuroblastoma cells. (**A**) IMR-5 cells were transfected with 50 nM of either WT-tRiMetF31 or Scr-tRiMetF31. Cells were treated with 10 μg/mL mitomycin C for 2 h prior to injury, and wound-healing assay was performed as described in Section 4. Data were expressed as mean ± SD for five migration images. (**B**) Tube formation assay was carried out with 50% conditioned medium from IMR-5 transfected with 50 nM of either WT-tRiMetF31 or Scr-tRiMetF31, as detailed in Section 4. Representative images were taken using a fluorescence microscope (100×). Data were expressed as mean ± SD for five images. (**C**) SK-N-AS cells were transfected with 50 nM of either WT-tRiMetF31 or Scr-tRiMetF31. Cells were treated with 10 μg/mL mitomycin C for 2 h prior to injury, and wound-healing assay was performed as described in Section 4. Data were expressed as mean ± SD for five migration images. (**D**) Tube formation assay was carried out with 50% conditioned medium from SK-N-AS cells transfected with 50 nM of either WT-tRiMetF31 or Scr-tRiMetF31 as detailed in Section 4. Representative images were taken using a fluorescence microscope (100×). Data were expressed as mean ± SD for five images. (**E**) IMR-5 cells were transfected with 50 nM of either tRiMetF31 siRNA or negative control siRNA. At 48 and 72 h after transfection, whole cellular lysates were prepared and subjected to Western blotting with antibody to PFKFB3, and GAPDH served as a loading control. (**F**) Tube formation assay was carried out with 50% conditioned medium from IMR-5 cells transfected with 50 nM of either tRiMetF31 siRNA or negative control siRNA as detailed in Section 4. Representative images were taken using a fluorescence microscope (100×). Data were expressed as mean ± SD for five images. *, *p* < 0.05; **, *p* < 0.01. Original western blot data is shown in Figure S3.

We next looked at the effect of tRiMetF31 on neuroblastoma cell migration and angiogenesis. Neuroblastoma IMR-5 and SK-N-AS cells were transfected with 50 nM of either WT-tRiMetF31 or Scr-tRiMetF31. Wound-healing assay showed that WT-tRiMetF31 had no effect on IMR-5 cell migration (Figure 6A), while it inhibited SK-N-AS cell migration (Figure 6C). Tube formation assay using a conditional medium from both lines indicated that WT-tRiMetF31 profoundly attenuated angiogenesis (Figure 6B,D). To further establish the crucial role of tRiMetF31/PFKFB3 axis in governing angiogenesis, tRiMetF31 was functionally inhibited by siRNA in IMR-5 cells. Western blot analysis showed that tRiMetF31 knockdown elevated the expression levels of PFKFB3 at 72 h after transfection (Figure 6E). As expected, tube formation assay indicated that tRiMetF31 siRNA significantly promoted angiogenesis (Figure 6F, *p* < 0.01). These results suggest a suppressive role of tRiMetF31 in neuroblastoma cell migration and angiogenesis through targeting PFKFB3.

## 3. Discussion

Despite the high relapse rate and poor prognosis of high-risk neuroblastoma, no curative treatment is available for patients with this disease. To our knowledge, this study for the first time demonstrated the anticancer property of CBN in neuroblastoma, highlighting a pivotal role of miR-34a/tRiMetF31/PFKFB3 axis in CBN-mediated antineuroblastoma signaling. The antitumor property of cannabinoids has long been known. In 1975, Munson AE and colleagues first reported the antineoplastic activity of Δ^8^-THC, Δ^9^-THC, and CBN in a Lewis lung adenocarcinoma mouse model [28]. The effects of CBD and THC on tumorigenesis were extensively investigated and indicated that they act in a CBR-dependent or -independent manner [29,30]. However, as a weak/nonpsychoactive cannabinoid, the impact of CBN on malignant diseases remains largely unknown. We show that CBN profoundly inhibits neuroblastoma cell proliferation, angiogenesis, and invasion in a dose-dependent manner, and induces cell cycle arrest through the inhibition of the AKT pathway and downregulation of miR-34a’s targets CDK2 and E2F1.

As a serine/threonine kinase, AKT (also known as PKB) is a major component of the PI3K/AKT pathway and plays a crucial role in numerous biological and pathologic processes. Overactivation of AKT is one of the most common molecular events driving the development of human malignancies [31,32], including neuroblastoma [16]. The PI3K/AKT pathway can be activated by numerous receptors anchored in cellular membranes, including ones for growth factors, cytokines, and chemokines. CBN, an agonist of CBRs with high affinity to CBR2 [33,34], remarkably increases CBR2 expression in SK-N-AS neuroblastoma cells, leading to inhibition of AKT pathway (Figure 2H), supporting a CBR2-dependent suppressive role of CBN in AKT signaling [35]. Interestingly, CBN also displays an inhibitory role in AKT pathway in IMR-5 neuroblastoma cells, despite it attenuating CBR2 expression (Figure 2G), which may implicate a receptor-independent suppression in AKT pathway. The signaling and crosstalk of ERK and AKT pathways are cell context-dependent, and they could activate or inhibit each other [36]. Although CBN inhibits the AKT pathway in the IMR-5 and SK-N-AS cell lines, this only leads to the activation of the ERK1/2 pathway in SK-N-AS cells and not IMR-5 cells (Figure 2G,H). Importantly, p53-binding protein MDM2 is a key target of AKT. Once it is phosphorylated by AKT, MDM2 translocates to the nucleus and triggers p53 degradation through binding [37]. In p53-wild-type IMR-5 cells, CBN causes a profound elevation in p53 protein (Figure 2G), which may imply a decrease in phosphorylated MDM2 due to the inhibition of the AKT pathway. As a transcription factor, tumor suppressor p53 transactivates numerous miRNAs, including the miR-34 family [38].

The miR-34 family is the most well-defined tumor suppressor microRNA family that consists of miR-34a, miR-34b, and miR-34c. A large number of oncogenes were identified to be direct targets of miR-34a [39] that contribute to the development of cancer hallmarks by promoting cell cycle, invasion, and angiogenesis, and suppressing apoptosis and differentiation, such as CDK2, cyclin E1, E2F1, notch1, and snail [39,40,41]. miR-34a is upregulated in neuroblastoma IMR-5 and SK-N-AS cells in response to CBN (Figure 2F), which may mechanically be through p53-dependent transactivation in IMR-5 and p53-independent-transactivation in SK-N-AS (Figure 2G,H) [38,42]. CBN also downregulates the expression of miR-34a targets CDK2, cyclin E1 and E2F1, supporting previous reports [39,40,41], which may contribute to CBN-mediated inhibition of proliferation, invasion, and angiogenesis, and S-phase cell cycle arrest (Figure 2A,B,D,E), because growing evidence demonstrates a pivotal role of E2F1 in these malignant processes [43,44]. However, our results do not suggest notch1 and snail as targets of miR-34a. miR-34a is key molecule mediating CBN’s antineuroblastoma effects, since CNB’s proliferative suppression is partially restored by miR-34a inhibitor (Figure 2I). Although this is the first report regarding the crucial role of miR-34a in CBN’s antineuroblastoma effects, miR-34a is downregulated in neuroblastoma cell lines and tissue [45], and neuroblastoma-targeted miR-34a nanoparticles significantly inhibited proliferation, angiogenesis, and neuroblastoma growth in an orthotopic mouse model [46].

Angiogenesis plays a key role in supporting tumor growth and metastasis. Two well-defined signaling pathways in regulating angiogenesis, VEGF and Notch, were extensively investigated [47], and numerous inhibitors targeting VEGF and Notch were developed and either approved by FDA or under Phase I clinical trials for cancer treatment [48,49]. One of the most attractive advances in angiogenesis is the recent discovery of PFKFB3-driven glycolysis in vessel sprouting [50]. PFKFB3 is an allosteric activator of 6-phosphofructo-1-kinse (PFK-1), which converts fructose-6-phosphate into fructose-1,6-biphosphate, a rate-limiting reaction of the glycolytic flux. PFKFB3 is linked to cancer and displays an oncogenic role in tumorigenesis. The elevated expression of PFKFB3 was reported in numerous human malignancies, including HER2+ breast cancer [51], head and neck squamous cell carcinoma [52], hepatocellular carcinoma [53], colon carcinoma [54], neuroblastoma [55], and pancreatic cancer [56], correlated with poor prognosis. Targeting PFKFB3 suppressed cancer cell proliferation in vitro and attenuated tumor growth or metastasis in animal models [51,52,53] and radiosensitized cancer cells [57]. In this study, miR-34a and its guided tRNA_i_^Met^-cleaved fragment tRiMetF31 were downregulated, whereas PFKFB3, a direct target of tRiMetF31, was overexpressed in all examined neuroblastoma cell lines (*n* = 4) (Figure 3B,D,E). The enforced overexpression of tRiMetF31 suppressed cell proliferation and angiogenesis, and induced the cell cycle arrest of neuroblastoma cell lines IMR-5 and SK-N-AS. Conversely, tRiMetF31 knockdown promoted PFKFB3 expression, consequently resulting in enhanced angiogenesis. Our findings revealed a key role of tRiMetF31, a novel component of the miR-34a tumor suppressor network, in suppressing proliferation and angiogenesis of neuroblastoma cells, supporting the application of target molecules miR-34a, tRiMetF31, and PFKFB3 in therapeutic intervention for neuroblastoma. Even though this is the first report of an inverse correlation between miR-34a/tRiMetF31 and PFKFB3 in neuroblastoma cell lines, it is essential to see the relationship in a large cohort of tissue sections. Unfortunately, we either did not have enough paired tissue samples for RNA isolation and cellular lysate preparation, or probe only recognizing/detecting tRiMetF31, not its parental mature tRNA_i_^Met^ and tRNA_i_^Met^ precursors. In the near future, with the advances in biotechnology, a tRiMetF31-specific probe may be successfully developed.

**Figure 7:**
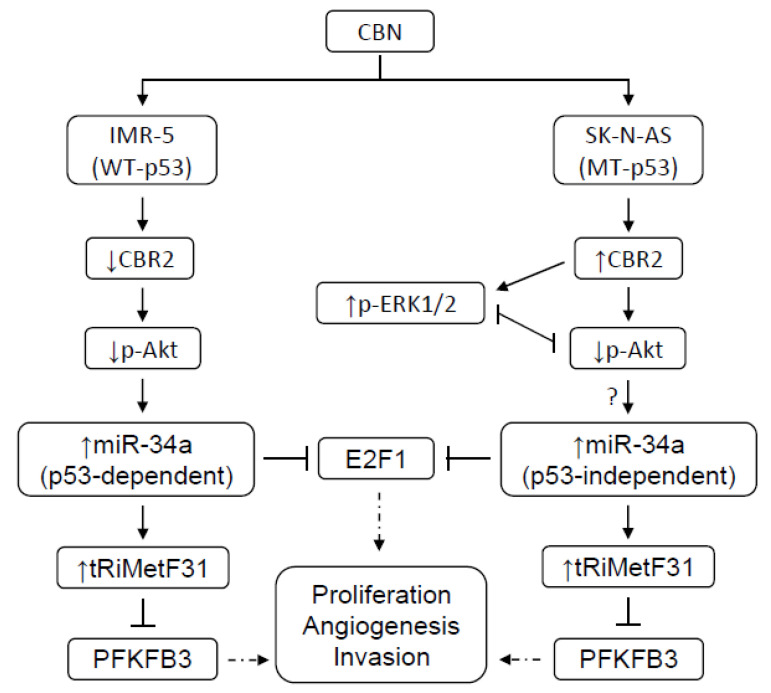
Proposed model for CBN’s anti-neuroblastoma mechanism. CBN’s antineuroblastoma effects may be mediated through CBR2-dependent and –independent mechanisms, leading to the inhibition of the AKT pathway and a p53-dependent or -independent transactivation of miR-34a. miR-34a, in turn, directly silences E2F1 and guides the production of tRiMetF31, which targets PFKFB3, eventually resulting in the suppression of neuroblastoma proliferation, invasion, and angiogenesis.

In conclusion, CBN may inhibit the AKT pathway by affecting CBR2 expression, leading to a p53-dependent or -independent transactivation of miR-34a; the latter directly silences E2F1 and guides the production of tRiMetF31 which targets PFKFB3, eventually resulting in suppression of neuroblastoma proliferation, invasion, and angiogenesis (Figure 7), highlighting novel and crucial tumor suppressor miR-34a/tRiMetF31/PFKFB3 axis in CBN’s antineuroblastoma actions. In the future, it would be important to further extend our findings. Our study serves as a roadmap for future in vivo animal model-based analysis.

## 4. Materials and Methods

### 4.1. Human Cell Lines

Foreskin fibroblast cell line BJ-5ta was cultured in Dulbecco’s Modified Eagle’s Medium (DMEM), and supplemented with 10% fetal bovine serum (FBS) and 1% penicillin/streptomycin (P/S). Embryonic kidney epithelial cell line HEK293, lung fibroblast cell line WI-38, neuroblastoma cell lines IMR-32 and SK-N-SH, and neuroepithelioma cell line SK-N-MC, were cultured in Eagle’s Minimum Essential Medium (EMEM) supplemented with 10% FBS and 1% P/S. Human mammary epithelial cell line HMEC was cultured in HuMEC Basal Serum Free Medium supplemented with HuMEC Supplement and Bovine Pituitary Extract and 1% P/S. Neuroblastoma cell line IMR-5, which was established from the tumor cells of a male 1-year-old child and was used to characterize the candidate genes associated with the pathogenesis of these tumors [58], was cultured in RPMI-1640 medium supplemented with 10% FBS, 1% nonessential amino acids (NEAA), and 1% P/S. Neuroblastoma cell line SK-N-AS, which was generated from the tumor of a 6-year-old female patient and was used as a model for neuroblastoma metastatic properties [59], was cultured in DMEM supplemented with 10% FBS, 1% nonessential amino acids (NEAA), and 1% P/S. Neuroblastoma cell lines SK-N-BE(2) and SH-SY5Y were cultured in a 1:1 mixture of EMEM and F12 medium supplemented with 10% FBS and 1% P/S. All cell lines were incubated at 37 °C in a humidified atmosphere of 5% CO_2_. Cell lines BJ-5ta, HEK293, HMEC, SH-SY5Y, SK-N-BE(2), and WI-38 were purchased from ATCC. Cell lines IMR-5, IMR-32, SK-N-AS, SK-N-MC, SK-N-SH were gift lines provided by Dr. Aru Narendran (Arnie Charbonneau Cancer Institute, University of Calgary, Calgary, AB, Canada). All cells were treated with mycoplasma removal reagent BM-Cyclin (Roche) to ensure negative mycoplasma before treatment.

### 4.2. Western Blot Analysis

The indicated cells grown to 85–95% confluency or at the indicated time-point after transfection were rinsed twice with ice-cold PBS and scraped off the plate in a radioimmunoprecipitation assay buffer (RIPA). Then, 50–100 μg of protein per sample was electrophoresed on 8% or 10% SDS-PAGE and electrophoretically transferred to a PVDF membrane (Amersham Hybond™-P, GE Healthcare, Chicago, IL, USA) at 4 °C for 1.5 h. Blots were incubated for 1 h with 5% nonfat dry milk to block nonspecific binding sites and subsequently incubated at 4 °C overnight with 1:200 to 1:1000 dilution of polyclonal/monoclonal antibodies against cannabinoid receptor 1 (CBR1) (from Abcam, Cambridge, UK) or CDK2, cyclin E1, E2F1, ERK1/2, notch1, pERK1/2, PFKFB3, snail (all from Cell Signaling, Danvers, MA, USA) or AKT1, cannabinoid receptor 2 (CBR2), p53, pAKT1/2/3 (all from Santa Cruz Biotechnology, Dallas, TX, USA). Immunoreactivity was detected using a peroxidase-conjugated antibody and visualized with an ECL Plus Western Blotting Detection System (GE Healthcare, Chicago, IL, USA). Blots were stripped before reprobing with antibody against GAPDH (Abcam, Cambridge, UK). The densitometry of bands was measured and normalized with that of GAPDH using ImageJ.

### 4.3. IC50 Determination 

IC50 is generally defined as half maximal inhibitory concentration; here, it represents the concentration of CBN suppressing 50% of cell growth. MTT-based IC50 measurement was performed as described previously [57,60] with some modifications. Briefly, IMR-5 and SK-N-AS cells grown to approximately 100% confluency in 96-well plate were exposed to a series concentration of CBN. At 72 to 96 h after incubation, 3-(4,5-dimethylthiazol-2-yl)-2,5-diphenyl tetrazolium bromide (MTT) assays were carried out using a Cell Proliferation Kit I (Roche Diagnostics GmbH, Mannheim, Germany) according to the manufacturer’s instructions. The spectrophotometric absorbance of samples was measured at 595 nm using a microtiter plate reader (FLUOstar Omega, Ortenberg, Germany). All MTT assays were performed in triplicate, and IC50 values were calculated using GraphPad Prism 8.2.1 software.

### 4.4. MTT Assay

In total, 3 × 10^3^ BJ-5ta, 1 × 10^3^ IMR-5, 2 × 10^3^ SK-N-AS, and 5 × 10^3^ WI-38 cells per well were plated in 96-well plate. At 24 h after plating, cells were treated with the indicated concentration of CBN. For miR-34a knockdown study, the IMR-5 cells grown to 80% confluency were transfected with 10 nM of either miRCURY LNA miR-34a Power Inhibitor (QIAGEN) or negative control A (QIAGEN) using Lipofectamine 3000 (ThermoFisher Scientific, Waltham, MA, USA) according to manufacturer’s instructions; at 24 h after transfection, 2000 cells per well were plated in 96-well plates and exposed to 15 μM CBN. For tRiMetF31 overexpression study, IMR-5 and SK-N-AS cells grown to 80% confluency were transfected with the indicated concentration of either wild-type tRiMetF31 (WT-tRiMetF31) or scrambled tRiMetF31 (Scr-tRiMetF31) using Lipofectamine 3000 (ThermoFisher Scientific) per manufacturer’s instructions; at 24 h after transfection, 3000 cells per well were plated in 96-well plates. The MTT assay was performed using a Cell Proliferation Kit I (Roche Diagnostics GmbH, Mannheim, Germany) according to the manufacturer’s instructions. The spectrophotometric absorbance of samples was measured at 595 nm using a microtiter plate reader (FLUOstar Omega, Ortenberg, Germany). All MTT assays were conducted in triplicate.

### 4.5. Cell Cycle and Apoptosis Analyses

IMR-5 and SK-N-AS cells grown to 85% confluency were exposed to either 15 or 20 μM CBN, or transfected with 50 nM of either WT-tRiMetF31 or Scr-tRiMetF31 using Lipofectamine 3000 (ThermoFisher Scientific) according to the manufacturer’s instructions. At 48 h after treatment or the indicated time-point after transfection (48 h or 72 h), cells were harvested for cell cycle and apoptosis analyses, which were carried out with a BD FACSCanto^TM^ II Flow Cytometer (BD Biosciences, Franklin Lakes, NJ, USA) using a propidium iodide staining solution and a BD Pharmingen^TM^ V-FITC Annexin Apoptosis Detection Kit (BD Biosciences) in triplicate, according to the manufacturer’s instructions.

### 4.6. Tube Formation Assay

Human umbilical vein endothelial cells (HUVECs) purchased from Invitrogen were cultured in Medium 200 containing Large Vessel Endothelial Supplement at 37 °C in a humidified atmosphere of 5% CO_2_. Angiogenesis assay was performed according to the manufacturer’s instructions. Briefly, Geltrex LDEV-Free Reduced Growth Factor Basement Membrane Matrix (Invitrogen, Grand Island, NY, USA) was thawed at 4 °C overnight; the thawed Geltrex matrix solution was mixed by pipetting up and down, and 0.1 mL of Geltrex Matrix was added to each well of a 24-well plate (prechilled at −20 °C) and incubated for 30 min at 37 °C. Cells were then harvested and resuspended to 1.9 × 10^5^ cells/mL in unsupplemented medium, and 0.25 mL of the cell suspension was combined with 0.25 mL of cancer cell-conditioned medium and slowly added to a precoated well. At 17 h after incubation, the HUVECs were stained with a cell-permeable dye, calcein, and visualized under a ZEISS fluorescence microscope (100×, Carl Zeiss Microimaging GmbH, Jena, Germany).

### 4.7. Cell Invasion Assay

Neuroblastoma cell invasion assay was performed using Cell Invasion Assay kit (Collagen I, Abcam) according to the manufacturer’s instructions with modifications. Briefly, top chambers were precoated with 100 μL of collagen I at 4 °C overnight; IMR-5 and SK-N-AS cells grown to 80% confluency were starved for 24 h in serum-free media; after starvation, cells were harvested and resuspended at 1 × 10^6^ cells/mL in serum-free medium; 600 μL of complete medium was added to the bottom chambers; 200 μL of cell suspension was gently mixed 100 μL of conditioned medium (Methanol, 15 μM CBN, and 20 μM CBN) and added to the top chambers, and incubated at 37 °C in CO_2_ incubator for 20 h or 48 h; after incubation, cells were fixed with 4% paraformaldehyde (PFA) for 20 min at room temperature; the uninvaded cells were removed carefully using cotton-tipped applicators; after air-dry, cells on the top chamber membrane were stained with 0.2% crystal violet for 10 min, and washed 3 times with distilled water; after air-drying, the membrane was mounted with clear nail polish. Invaded cells were observed under an inverted microscope (ZEISS, 200×).

### 4.8. Quantitative Real-Time RT-PCR (qRT-PCR)

To measure hsa-miR-34a levels, total RNA either isolated from the indicated cells using TRIzol reagent (Invitrogen) or purchased from BioChain (Total RNA of human adult normal brain tissue, Newark, CA, USA) was subjected to qRT-PCR using miScript II RT Kit and QuantiTect SYBR Green PCR Master Mix with a primer set for hsa-miR-34a (QIAGEN, Germantown, MD, USA) according to the manufacturers’ instructions. Human *RNU6-2* served as loading control. To determine the total and precursor tRNA_i_^Met^ levels, total RNA was isolated using TRIzol reagent (Invitrogen), and qRT-PCR was performed as described previously [27]. Experiments were performed in triplicate. To measure tRiMetF31 levels, total RNA was isolated using TRIzol reagent (Invitrogen), and qRT-PCR was performed as described previously [61]. Briefly, 6 μg of total RNA samples were separated by electrophoresis on a 15% denaturing polyacrylamide gel (1× DEPC-treated TBE buffer, 15% acrylamine/bis-acrylamide (19:1), 6.5 M urea, 0.08% ammonium persulfate, 0.04% N,N,N′,N′-tetramethyl-ethylenediamine) 80 V for 4 h; after electrophoresis, gel was stained with Safe-Red (Applied Biological Materials Inc., Richmond, BC, Canada), and RNA was visualized under UV; a piece of gel containing 15-150 nt RNA was cut off, and the small RNA was purified using ZR small-RNA PAGE Recovery Kit (ZYMO Research, Irvine, CA, USA) according to the manufacturer’s instruction. The recovered small RNA was then polyadenylated using poly(A) polymerase (Ambion, Austin, TX, USA) and purified with TRIzol reagent (Invitrogen) per the manufacturer’s instructions. Reverse transcription for 31 nt tRiMetF31 qPCR was performed using iScript^TM^ Select cDNA Synthesis kit (Bio-Rad, Hercules, CA, USA) with RTQ primer [61], and qRT-PCR was carried out using SsoFast^MT^ EvaGreen Supermix (Bio-Rad) with tRiMetF-SP and RTQ-UNIr primers according to the manufacturer’s instructions. The reverse transcription for RNU6-2 loading control was performed using miScript II RT Kit (QIAGEN), and qRT-PCR was then performed using miScript SYBR^®^ Green PCR Kit (QIAGEN) with RNU6-2 primer set per the manufacturer’s instructions. Primers used in this study were RTQ primer: 5′-CGA ATT CTA GAG CTC GAG GCA GGC GAC ATG GCT GGC TAG TTA AGC TTG GTA CCG AGC TCG GAT CCA CTA GTC CTT TTT TTT TTT TTT TTT TTT TTT TTG C-3′; tRiMetF31 specific primer (tRiMetF-SP): 5′-AAG CGT GCT GGG CAA AAA-3′; RTQ-UNIr: 5′-CGA ATT CTA GAG CTC GAG GCA GG-3′.

### 4.9. Wound Healing Assay

IMR-5 and SK-N-AS cells grown to 80% confluency were transfected with 50 nM of either WT-tRiMetF31 or Scr-tRiMetF31 using Lipofectamine 3000 (ThermoFisher Scientific) according to the manufacturer’s instructions. At 24 h after transfection, cells were replated in 6-well plates and incubated for another 24 h. Cells were treated with 10 μg/mL mitomycin C (Sigma, Burlington, MA, USA) for 2 h prior to injury, and wound-healing assay was performed as described previously [62].

### 4.10. Statistical Analysis

Student’s *t*-test was used to determine the statistical significance of the differences in CBR1 and CBR2, PFKFB3, hsa-miR-34a, and tRiMetF13 and tRNA_i_^Met^ expression, cell growth, cell migration, invasion, tube formation, apoptosis, and cell cycle among groups. Apoptosis and cell cycle analyses were conducted in duplicate, while other experiments were performed in triplicate. *p* < 0.05 was considered to be significant.

## Data Availability

All data needed to evaluate the conclusions of this paper are present in the paper and/or supplementary materials. Additional data related to this paper may be requested from the authors.

## Supplementary Materials

The following supporting information can be downloaded at: https://www.mdpi.com/article/10.3390/cancers14081908/s1, Figure S1: CBN IC50 measurement. Figure S2: Effect of tiMetF31-induced downregulation of PFKFB3 on proliferation of neuroblastoma IMR-32 cells. Figure S3: Original western blot data for the manuscript.
