# Supplementary material for: Cannabinol Inhibits Cellular Proliferation, Invasion, and Angiogenesis of Neuroblastoma via Novel miR-34a/tRiMetF31/PFKFB3 Axis
Source: Cancers (Basel). 2022 Apr 10;14(8):1908. doi: 10.3390/cancers14081908 (PMC9027424; doi:10.3390/cancers14081908)
Supplement: Supplementary file 1 [file cancers-14-01908-s001.zip › cancers-1652842-Supplementary.pdf]

Supplementary Materials: Cannabinol Inhibits Cellular Proliferation, Invasion, and Angiogenesis of Neuroblastoma via Novel miR-34a/tRiMetF31/PFKFB3 Axis

Bo Wang, Dongping Li, Viktoriia Cherkasova, Marta Gerasymchuk, Aru Narendran, Igor Kovalchuk and Olga Kovalchuk

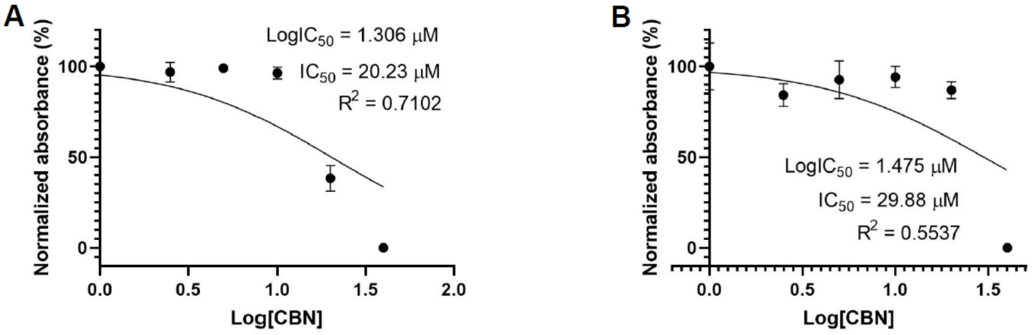

Figure S1. CBN IC50 measurement. IMR-5 (**A**) and SK-N-AS (**B**) cells grown to approximately 100% confluency in 96-well plate were exposed to a series concentration of CBN. At 72 h to 96 h after incubation, the MTT assays were carried out as detailed in the "Methods". All MTT assays were done in triplicate, the IC50 values were calculated using GraphPad Prism 8.2.1 software.

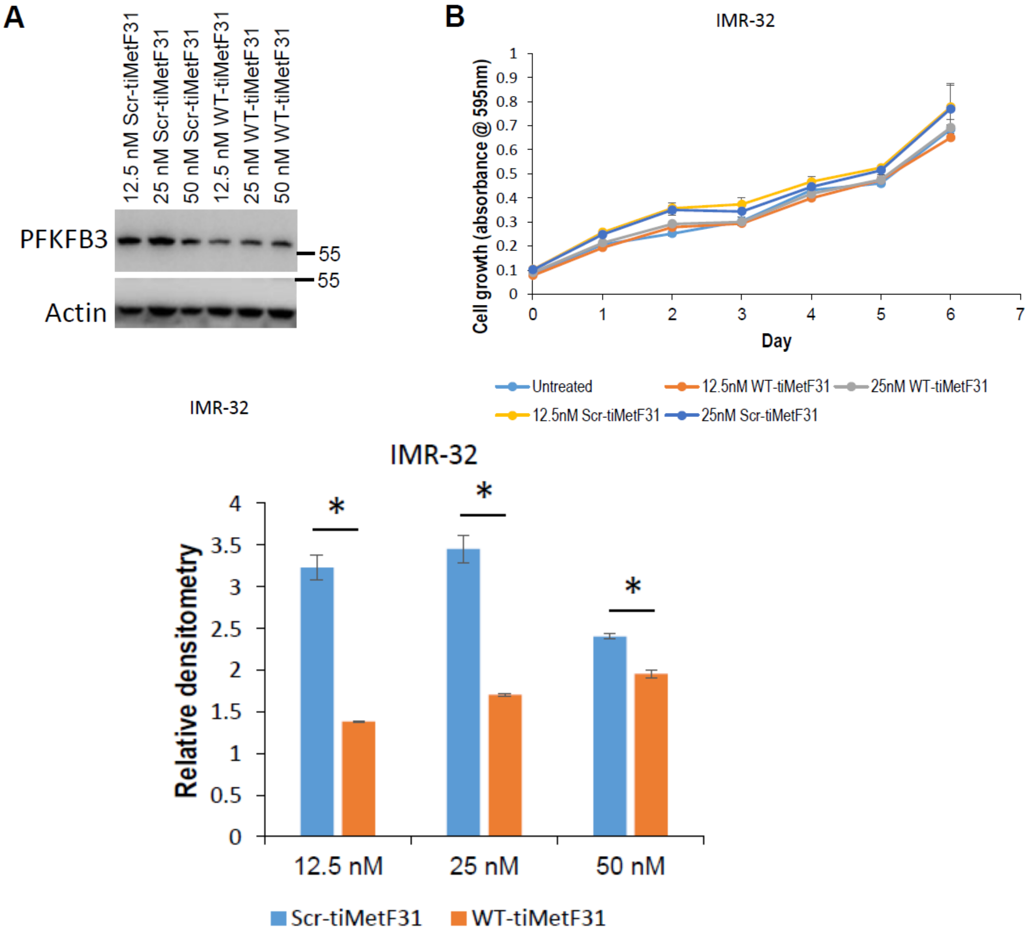

Figure S2. Effect of tiMetF31-induced downregulation of PFKFB3 on proliferation of neuroblastoma IMR-32 cells. IMR-32 cells grown to 80% confluency were transfected with the indicated

concentration of either WT-tiMetF31 or Scr-tiMetF31. (**A**) At 48 h after transfection, whole cellular lysates were prepared and subjected to Western blotting with antibody to PFKFB3, GAPDH served as a loading control. Relative densitometry was measured using ImageJ, calculated as a ratio to GAPDH and expressed as mean ± SD for three independent measurements. (**B**) At 24 h after transfection, 3,000 cells per well were replated in 96-well plates, the MTT assay was performed as described in the "Methods". * indicates $p < 0.05$.

Original Western blotting images: Figure 1A

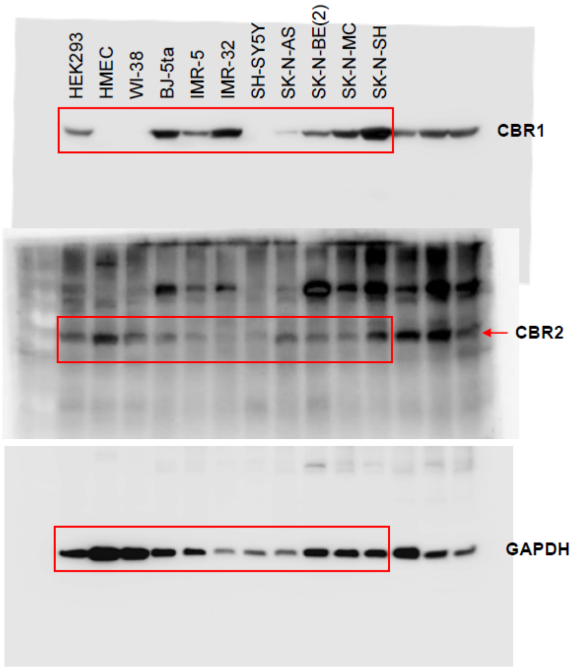

Original Western blotting images: Figure 2G and H

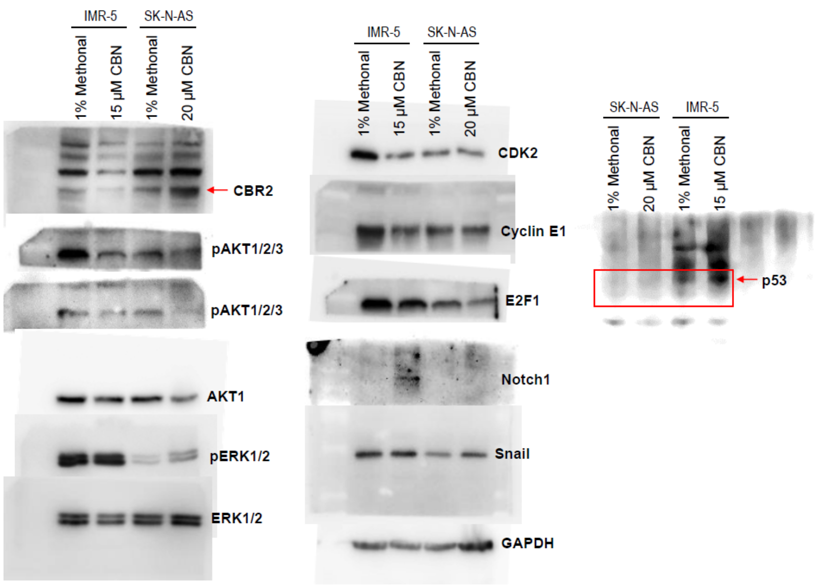

Original Western blotting images: Figure 2J

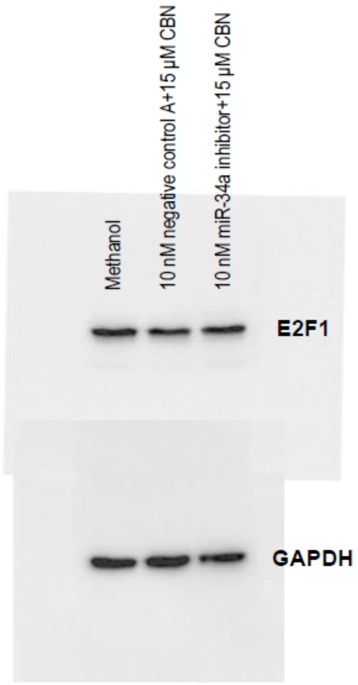

Original Western blotting images: Figure 3E

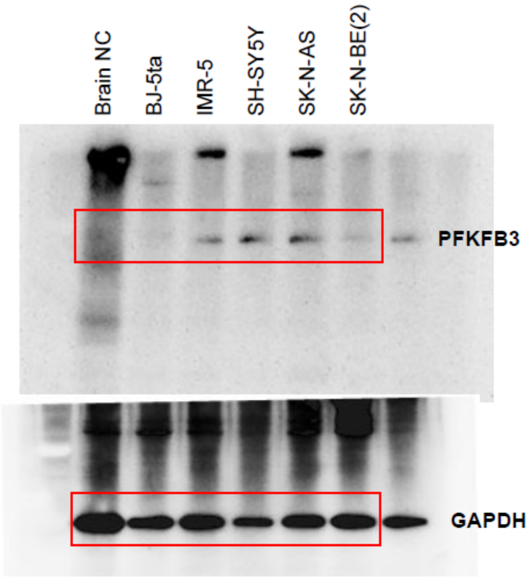

Original Western blotting images: Figure 4A

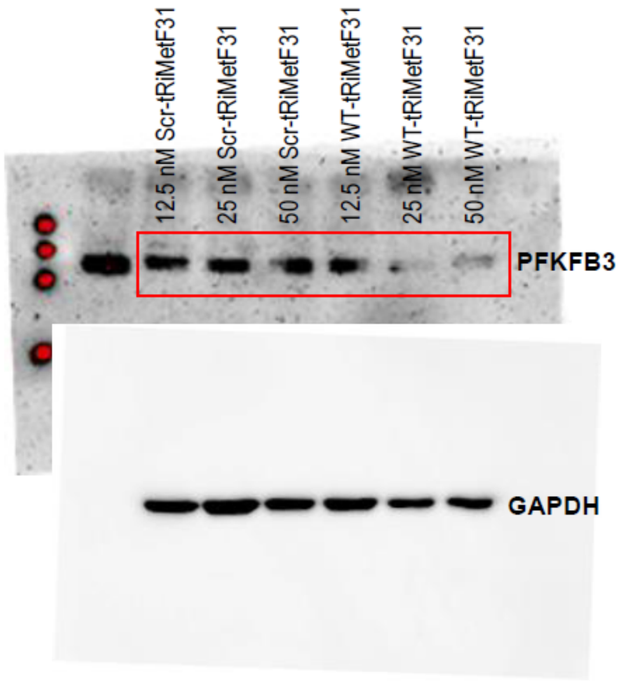

Original Western blotting images: Figure 5A

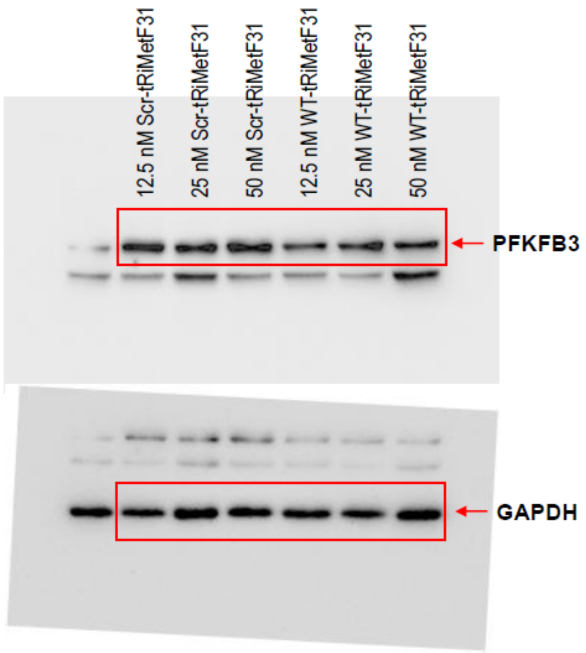

Original Western blotting images: Figure 6E

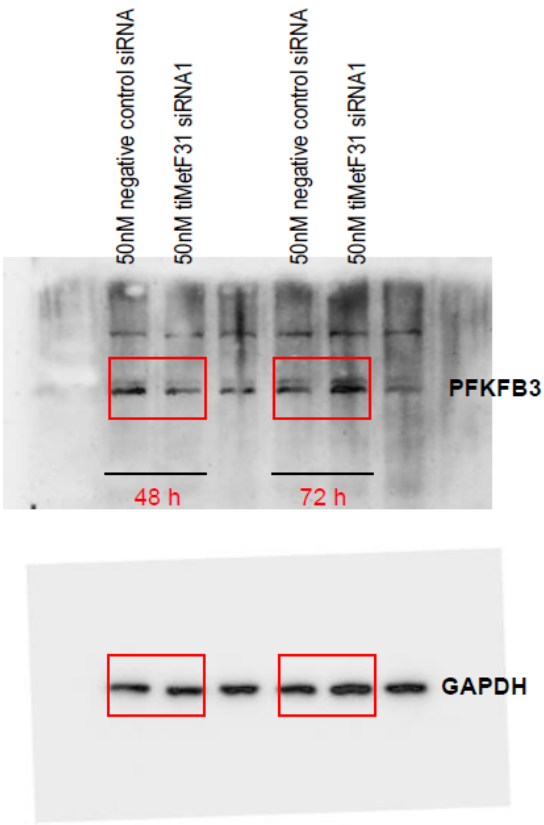

Original Western blotting images: Figure S2A

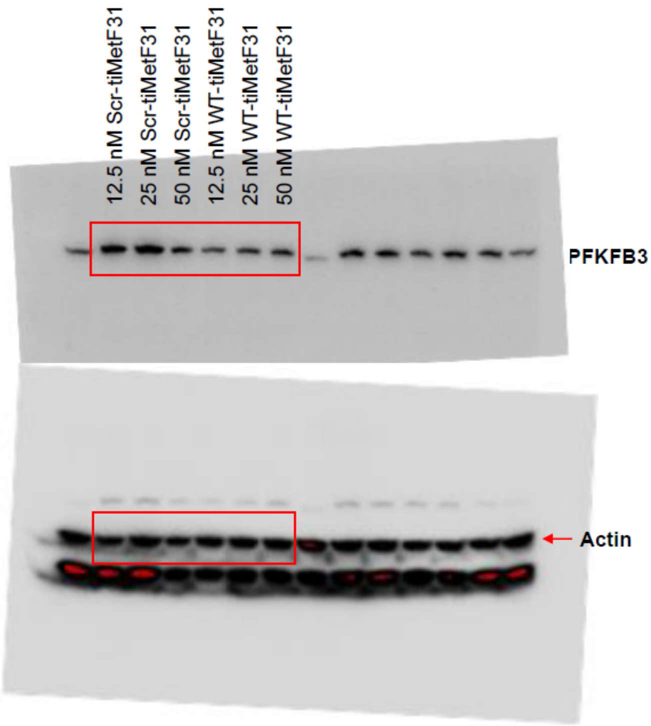

Figure S3. Original western blot data for the manuscript.
